# Supplementary figures and images for: Nonsterile immunity to cryptosporidiosis in infants is associated with mucosal IgA against the sporozoite and protection from malnutrition
Source: PLoS Pathog. 2021 Jun 28;17(6):e1009445. doi: 10.1371/journal.ppat.1009445 (PMC8270466; doi:10.1371/journal.ppat.1009445)

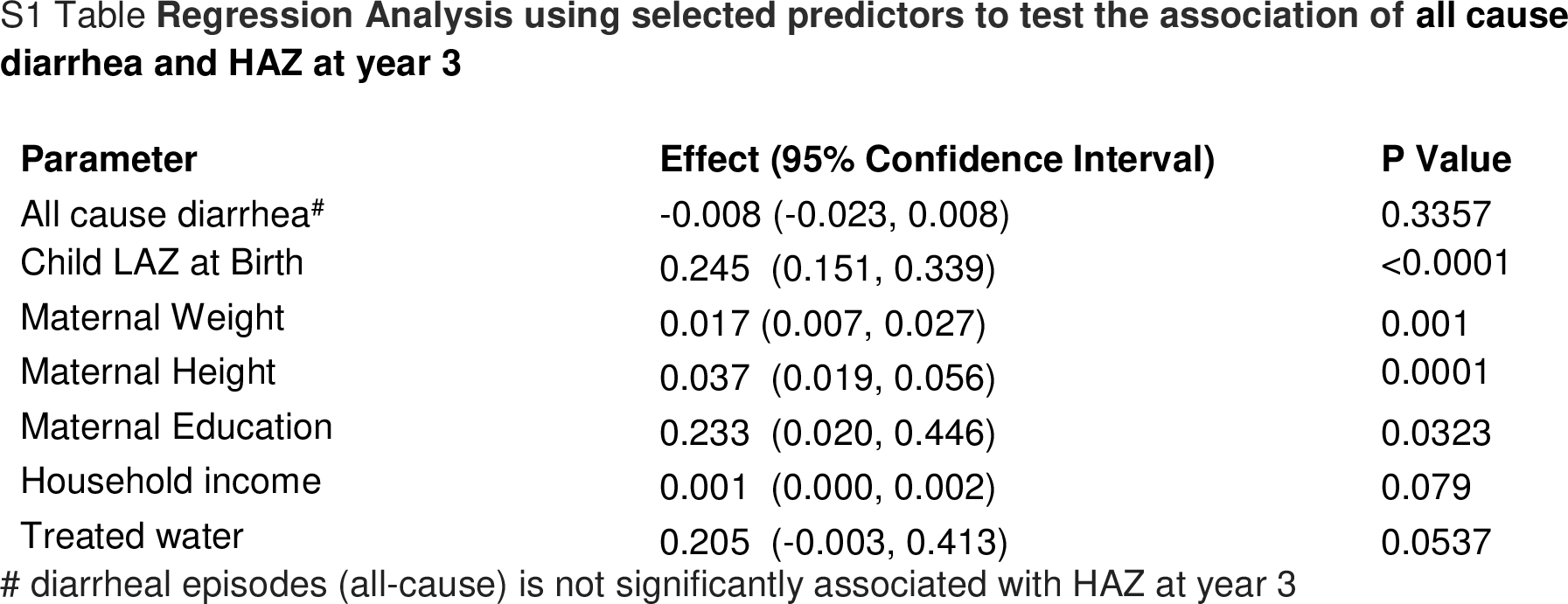

Supplement: S1 Table — (TIF) [file ppat.1009445.s001.tif]

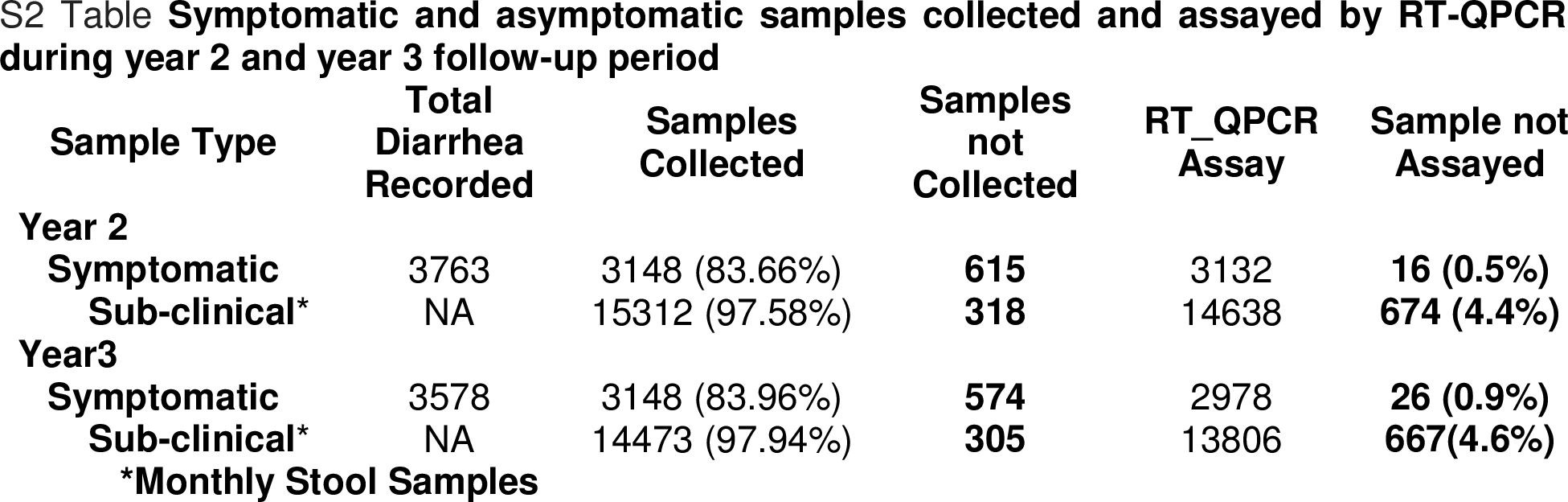

Supplement: S2 Table — (TIF) [file ppat.1009445.s002.tif]

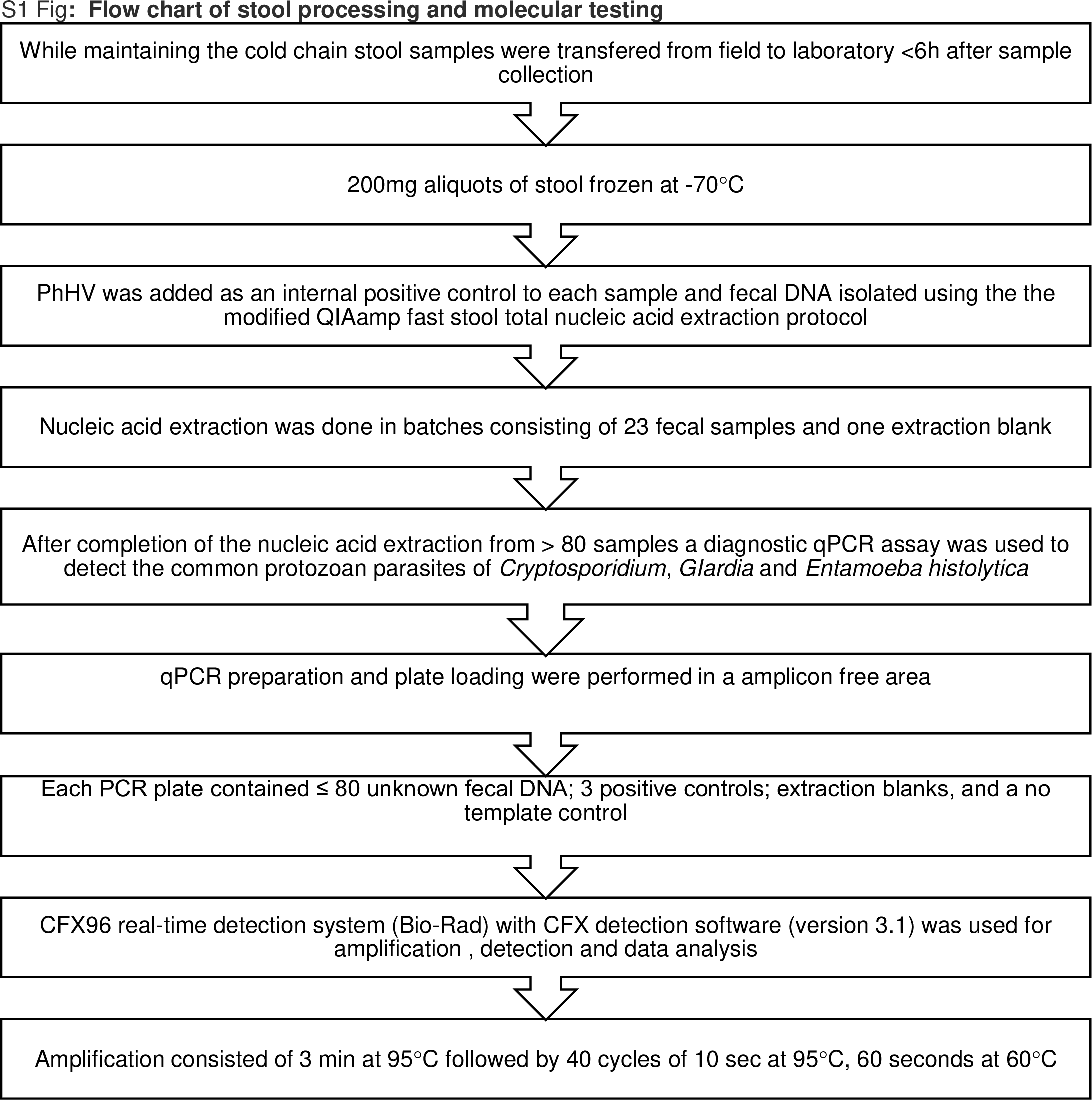

Supplement: S1 Fig — (TIF) [file ppat.1009445.s003.tif]

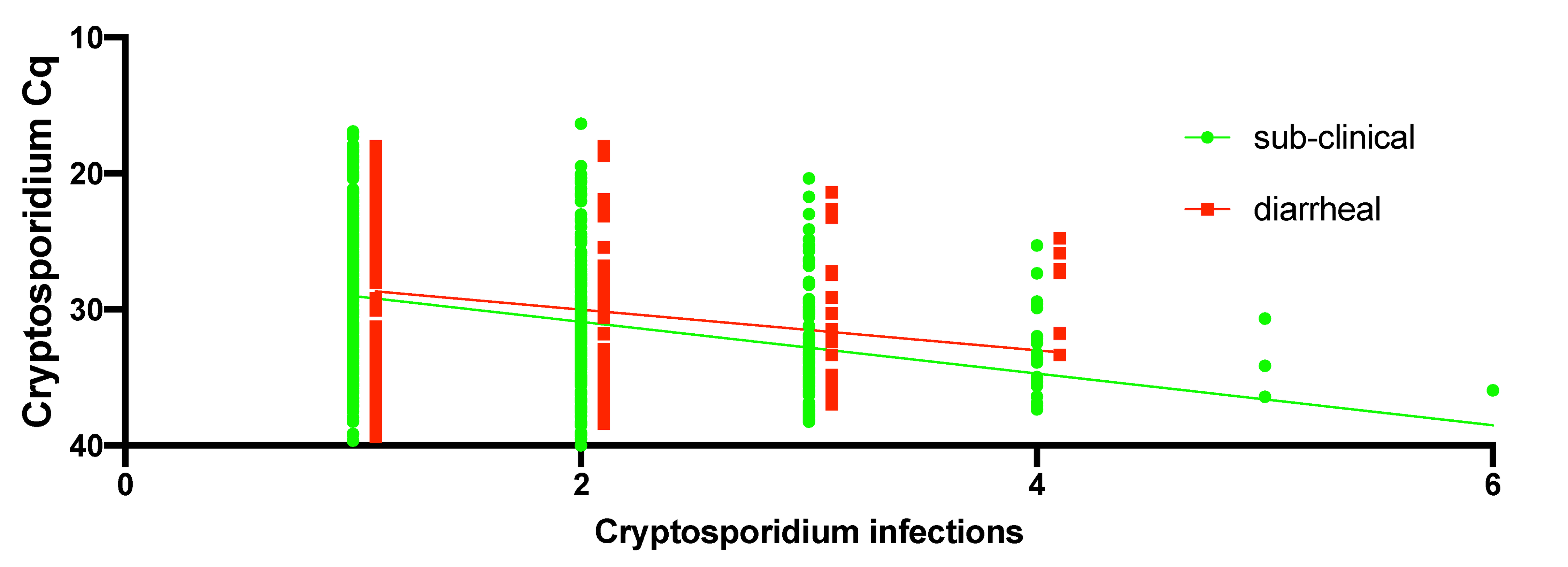

Supplement: S2 Fig — Relationship between Parasite Burden and the number of recurrent Cryptosporidium infections. Each symbol represents the first detectable sample of an individual infection. Y-axis, quantitative cycle of the diagnostic pan-Cryptosporidium PCR assay (Cq). X-axis, the total number of Cryptosporidium infections. The infection was designated as either diarrheal (red) or sub-clinical (green) based on the current infection phenotype. The data from diarrheal cases was offset to improve data visualization. To account for within-child correlations among repeated Cryptosporidium infections, the generalized estimating equation (GEE) method for repeated measurements were used with exchangeable correlation structure. As the intercept of the diarrheal and sub-clinical models was not statistically different the common intercept (27.02 ± 0.45) was used. The slope of the data derived from the sub-clinical (1.9 ± 0.2) and diarrheal (1.49 ± 0.31) exchangeable models were not significantly different from each other (p = 0.071) although both were statistically different from zero (p<0.0001). (TIF) [file ppat.1009445.s004.tif]

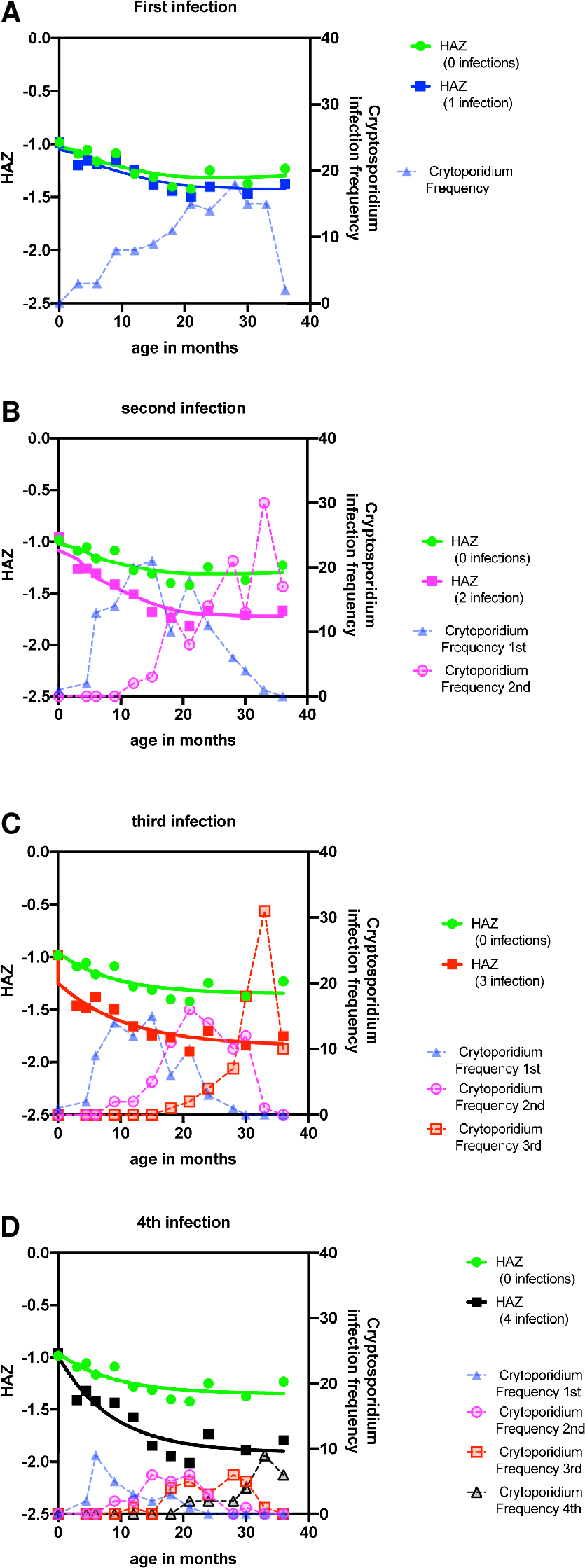

Supplement: S3 Fig — x axis child age in months; left y-axis child HAZ scores; right y-axis frequency of Cryptosporidium (diarrheal and sub-clinical) infections (shown as the number that occurred per the age of the child in months). All graphs include as a reference the HAZ score of children where no Cryptosporidium infections were detected (green circle and line). Cryptosporidium infections: Light blue triangle dotted blue connection line: infection one; purple circle and dotted line: infection two; light red square and dotted line: infection three; black triangle and dotted line: infection four A) blue symbol and solid line HAZ score of children who had one Cryptosporidium infections by 3 years of age B) purple square and solid line HAZ score of children who had two Cryptosporidium infections by three years of age C) red square and solid line HAZ score of children who had three Cryptosporidium infections by three years of age D) black square and solid line HAZ score of children who had four Cryptosporidium infections by three years of age. (TIF) [file ppat.1009445.s005.tif]

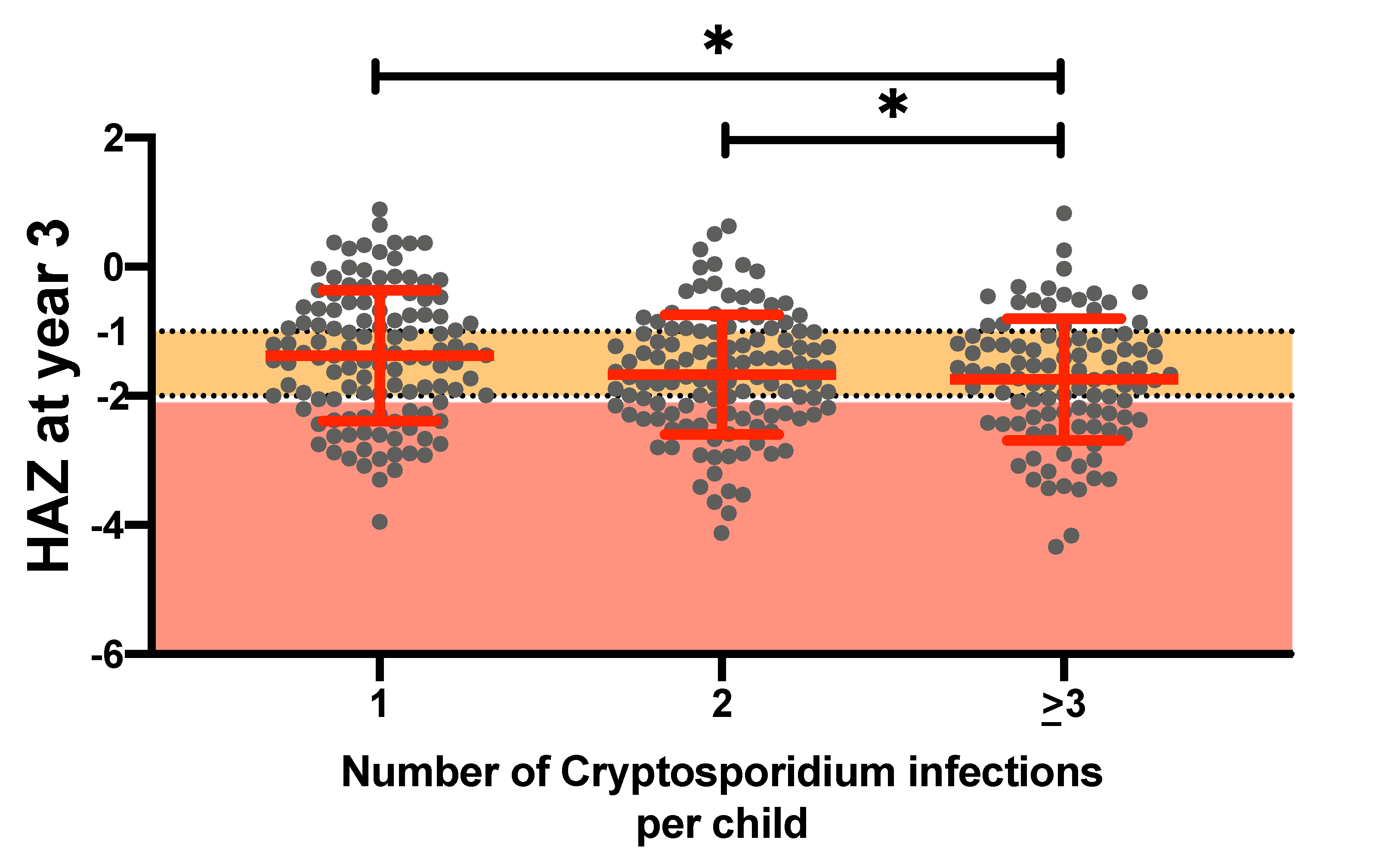

Supplement: S4 Fig — Box plot comparing the height for age z score at 3 years (HAZ) (Y-axis) mean and standard deviation shown Children were considered to be at Risk for malnutrition is they have a HAZ score <-1 and malnourished at HAZ-2: orange box: 3-year HAZ score -1 to -2; red box 3-year HAZ score < -2. X-axis Number of Cryptosporidium infections. Bar indicates the result of a non-parametric Kruskal-Wallis test for multiple comparisons * indicates p<0.05 ** indicates p<0.01. (TIF) [file ppat.1009445.s006.tif]

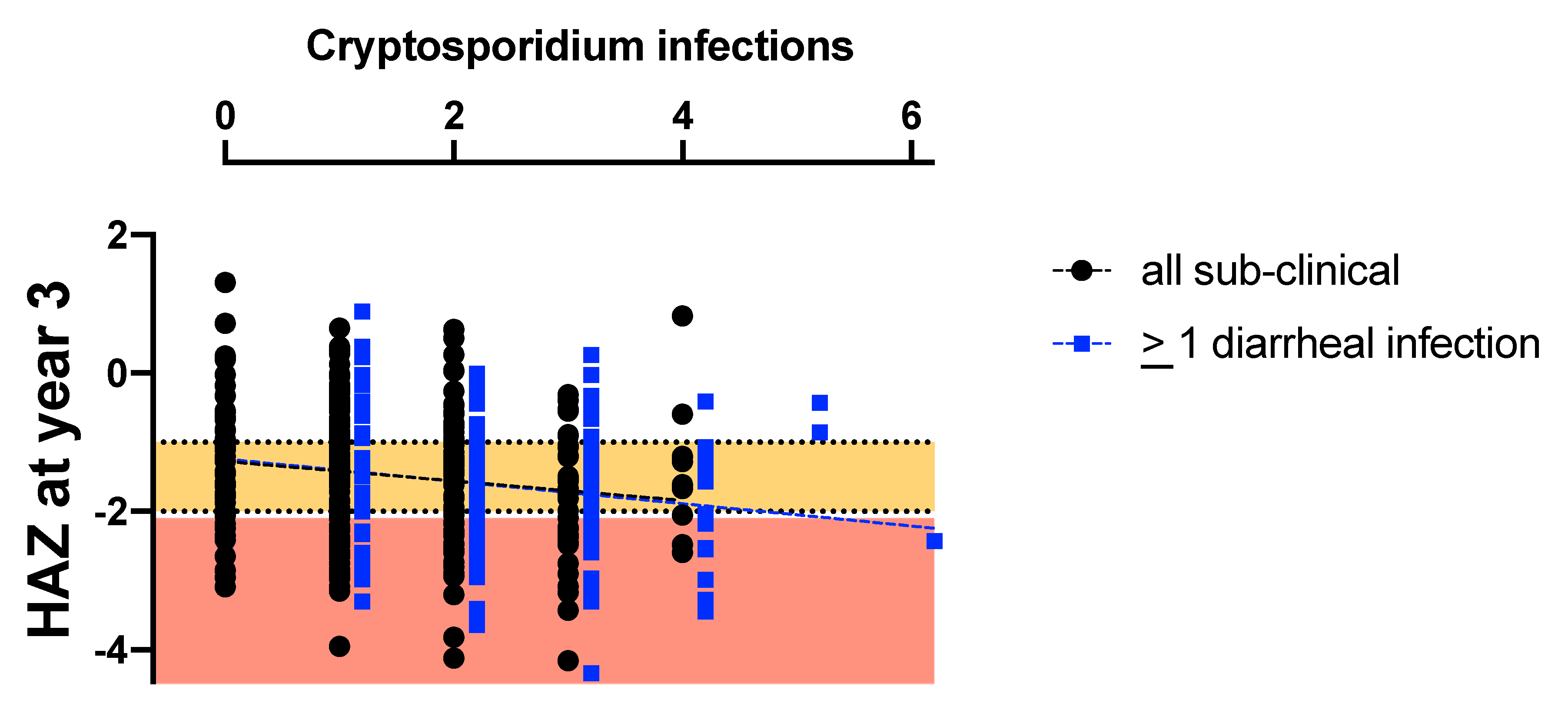

Supplement: S5 Fig — Graphs show results from a simple linear regression with each symbol representing a single child. Black symbols represent children who were never infected or had sub-clinical infections. The blue symbols indicate children who have had one or more than one episodes of diarrhea- associated cryptosporidiosis. Height for age (HAZ) z score at 3 years is shown on the Y-axis. The slope of the diarrheal-associated and sub-clinical groups are identical. Pooled Slope: -0.1545. Children are considered to be at Risk for malnutrition if they have a HAZ score <-1 and malnourished at HAZ -2: orange box: 3-year HAZ score -1 to -2; red box: 3-year HAZ score < -2. X-axis indicates number of Cryptosporidium infections. (TIF) [file ppat.1009445.s007.tif]

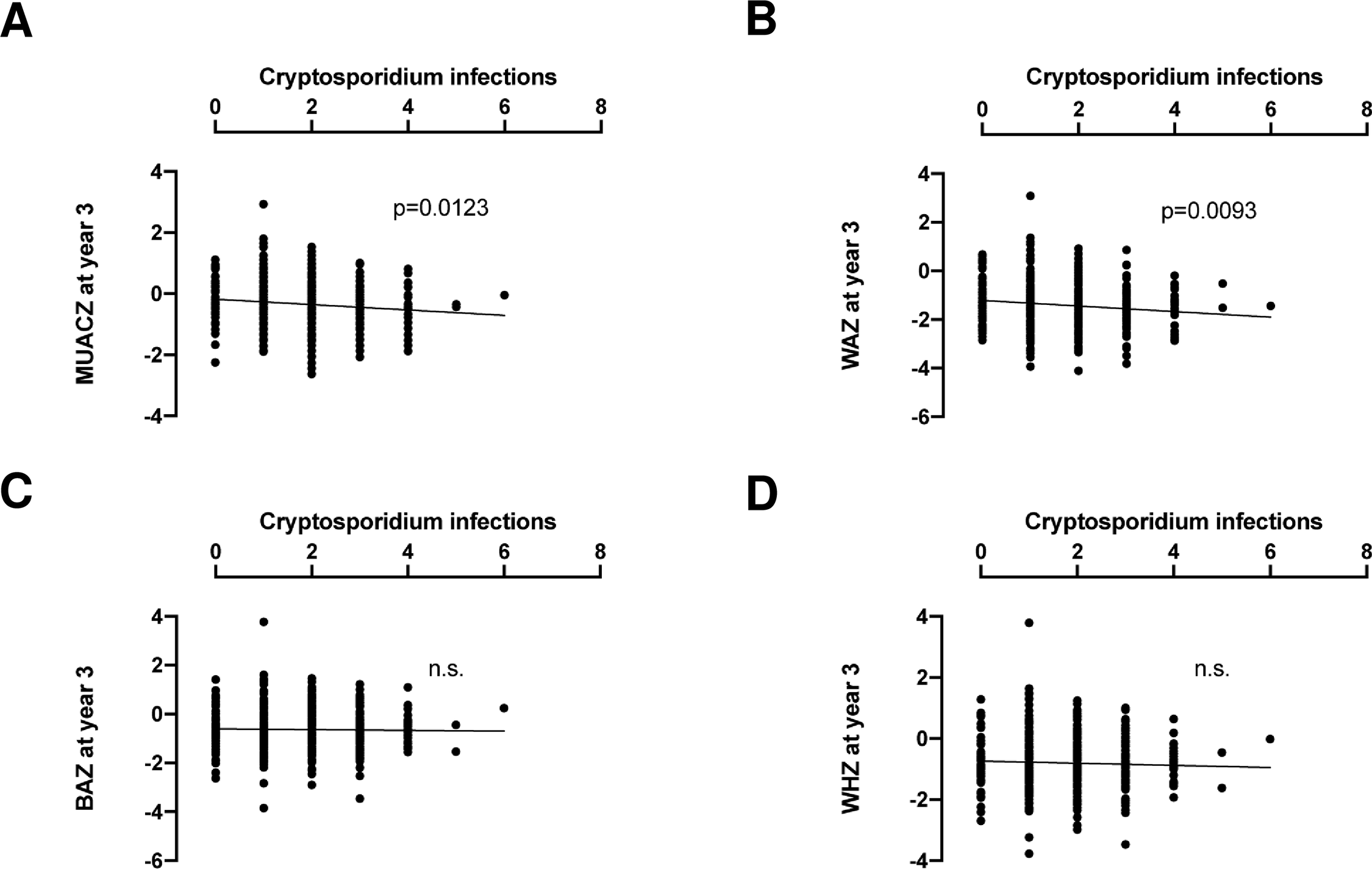

Supplement: S6 Fig — Graphs show results from a simple linear regression with each symbol representing a single child X-axis indicates number of Cryptosporidium infections A) Y-axis MUACZ circumference of the mid-upper arm (muscle wasting) B) Y-axis WHZ score (low weight for height (wasting) a measure of acute malnutrition C) Y axis WAZ score (low weight for age) a measure of acute and chronic malnutrition and D) BAZ (body mass index for age). (TIF) [file ppat.1009445.s008.tif]

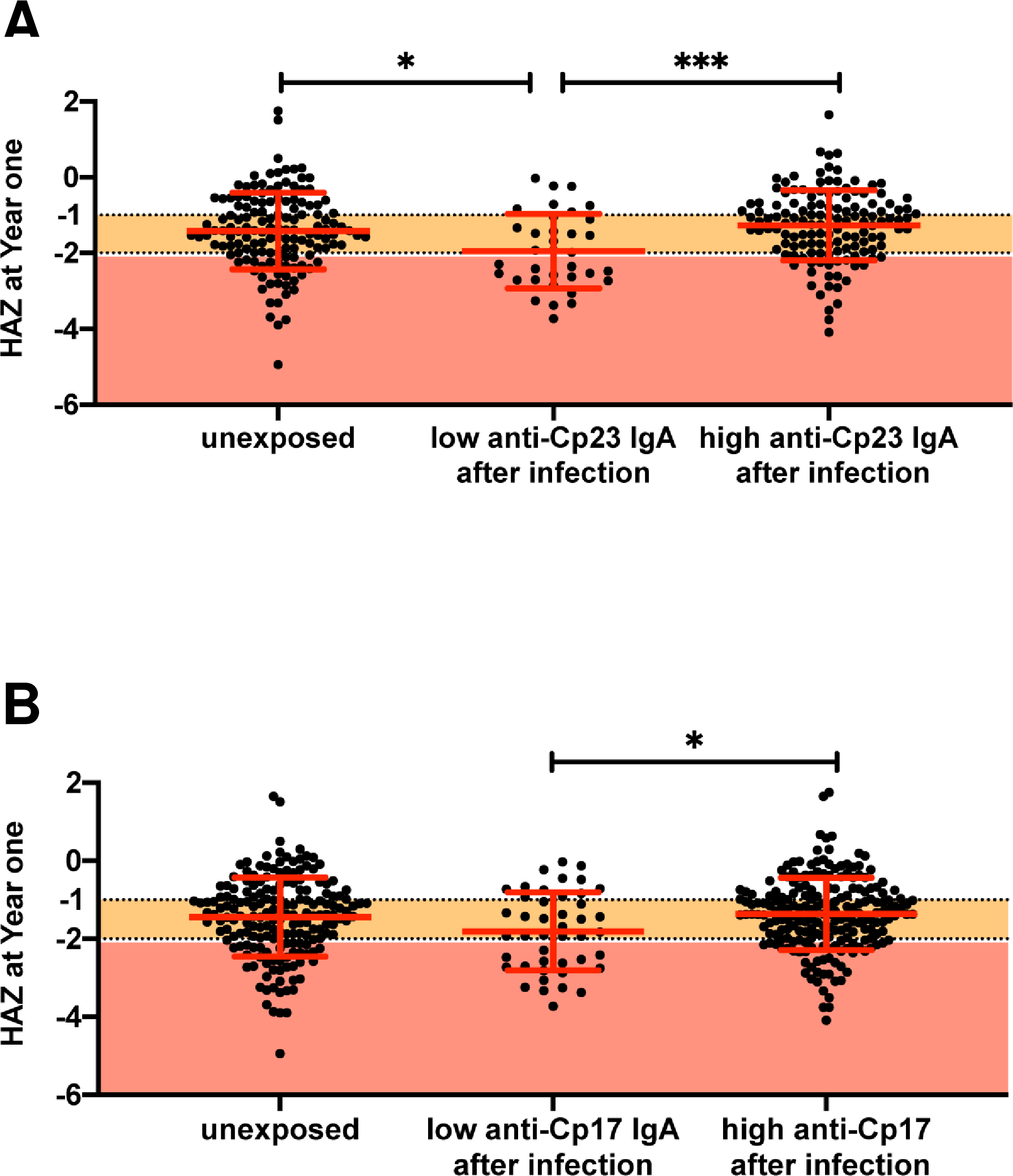

Supplement: S7 Fig — (TIF) [file ppat.1009445.s009.tif]

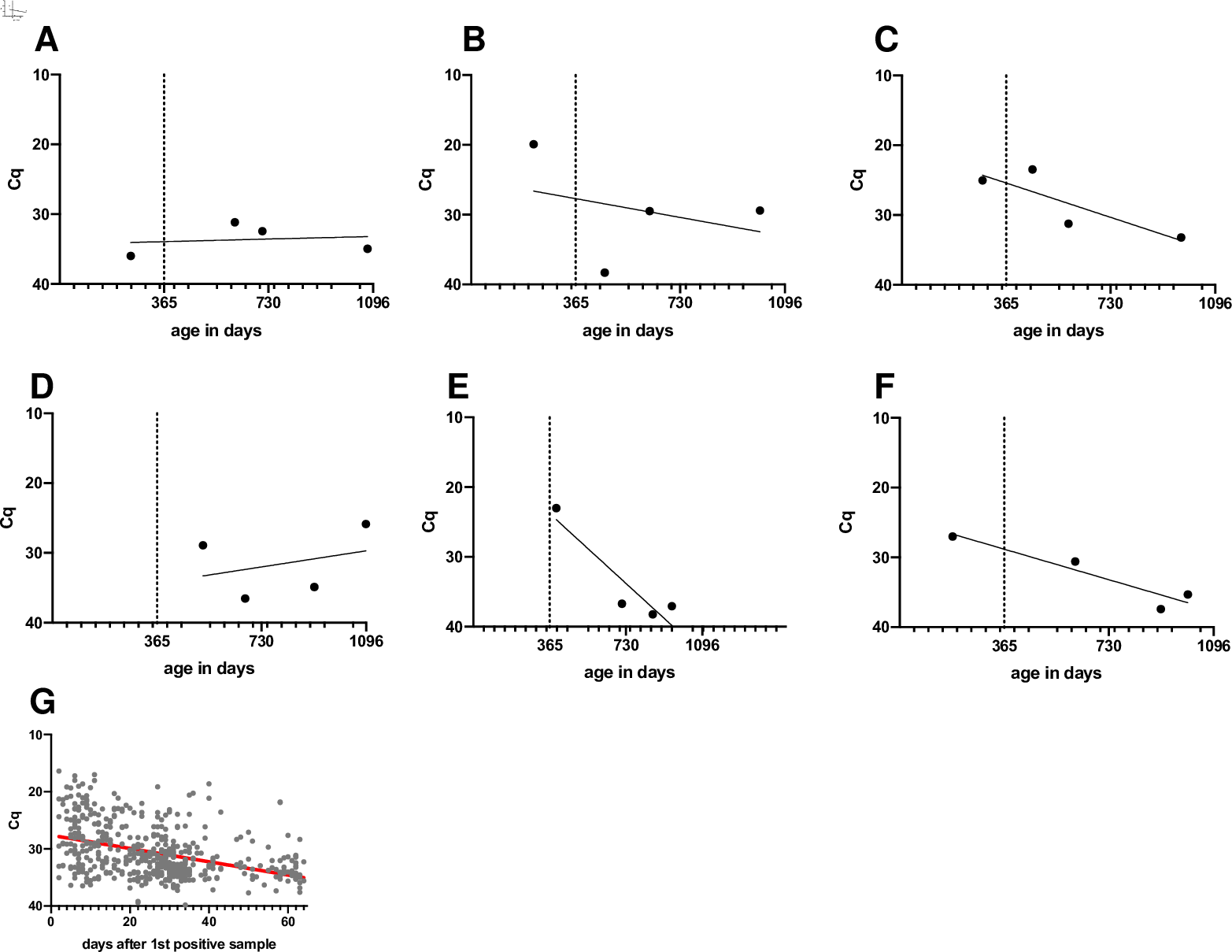

Supplement: S8 Fig — Each symbol represents the first detectable sample of an individual infection. Y-axis, quantitative cycle of the diagnostic pan-Cryptosporidium PCR assay (Cq). X-axis age of child in days A-C Infants with high fecal IgA anti-Cryptosporidium at year one D-F Infants with low fecal IgA anti-Cryptosporidium at year one G). (TIF) [file ppat.1009445.s010.tif]

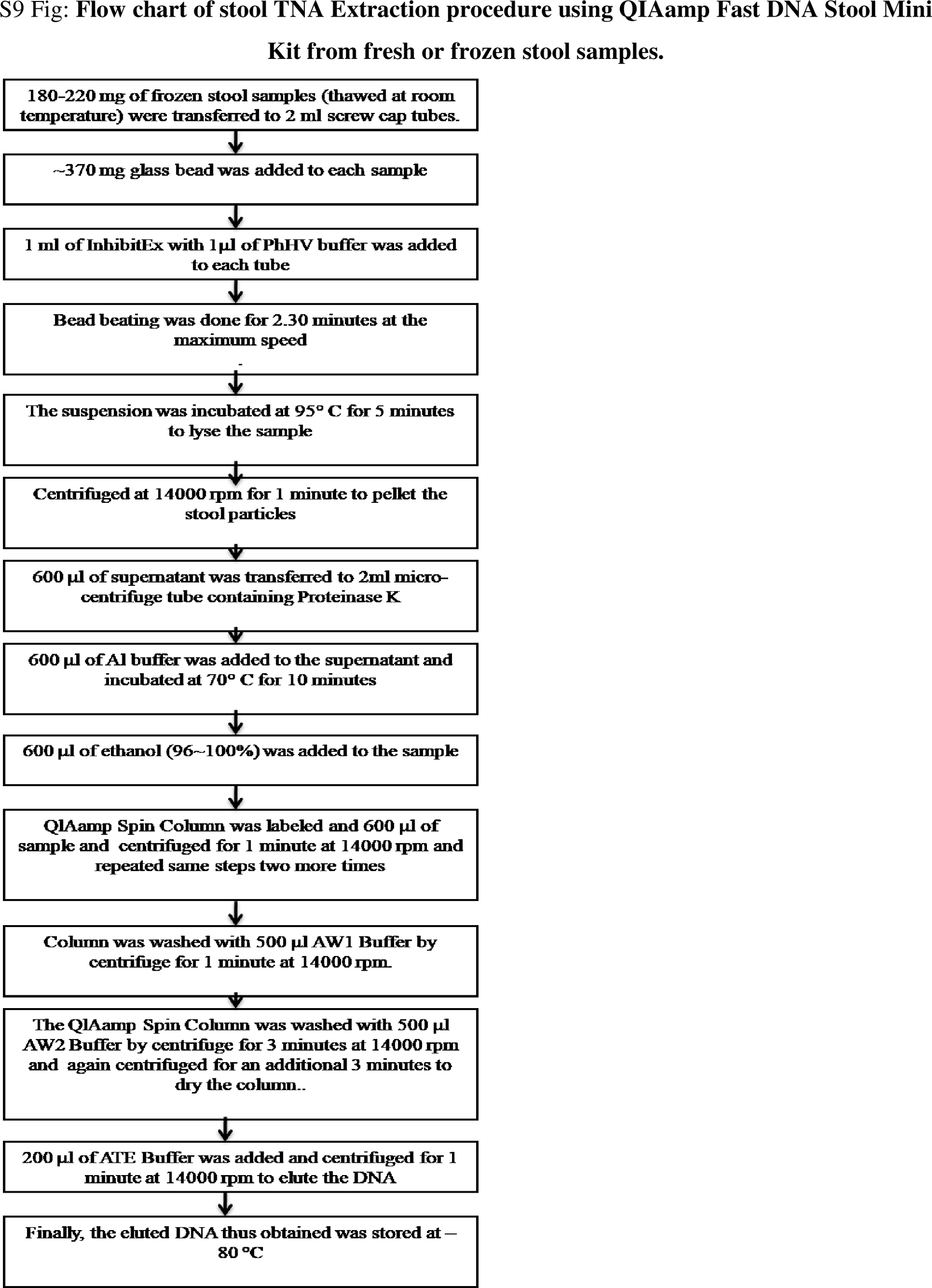

Supplement: S9 Fig — (TIF) [file ppat.1009445.s011.tif]

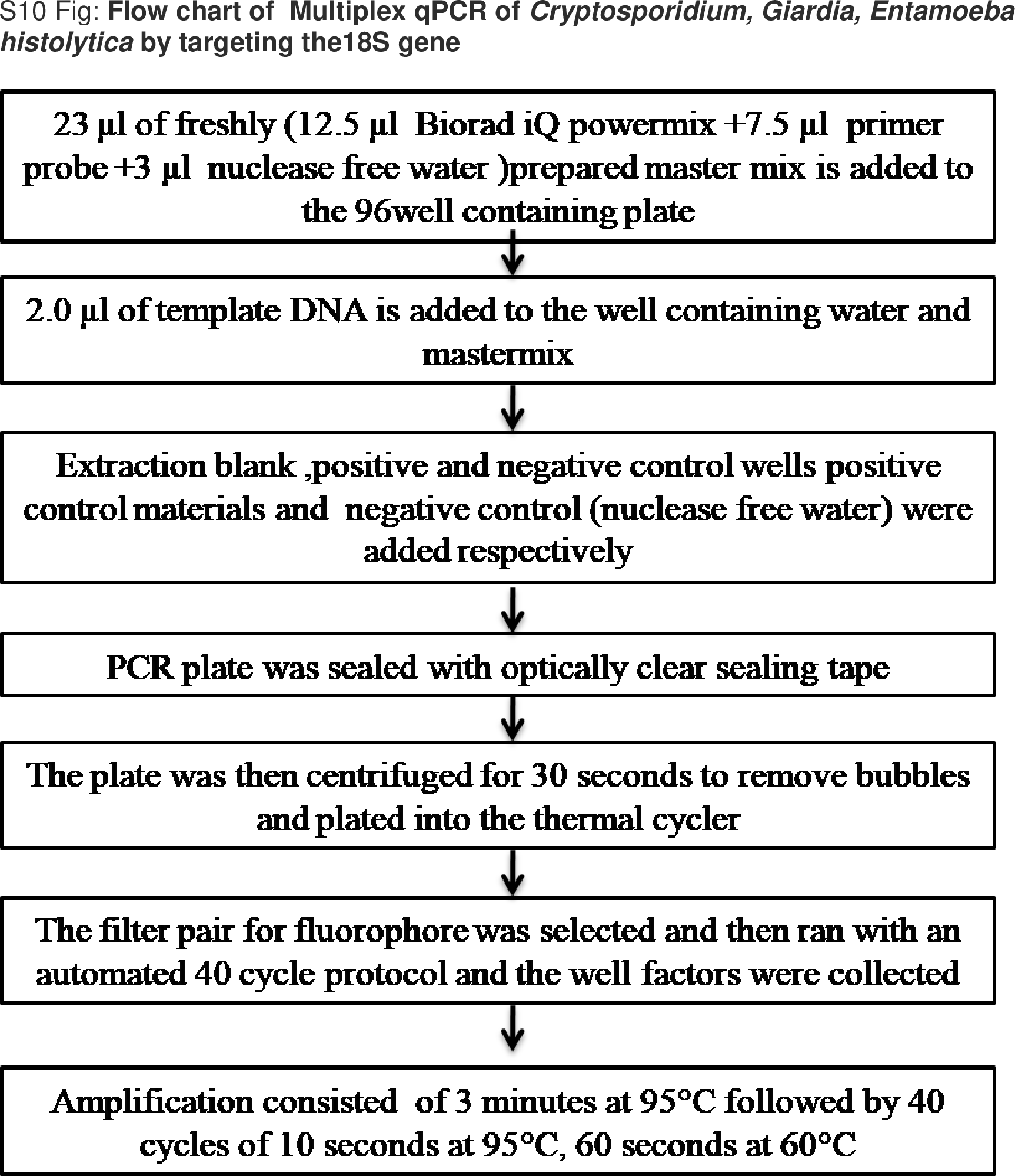

Supplement: S10 Fig — (TIF) [file ppat.1009445.s012.tif]
