# Supplementary material for: Nonsterile immunity to cryptosporidiosis in infants is associated with mucosal IgA against the sporozoite and protection from malnutrition
Source: PLoS Pathog. 2021 Jun 28;17(6):e1009445. doi: 10.1371/journal.ppat.1009445 (PMC8270466; doi:10.1371/journal.ppat.1009445)
Supplement: Attachment — Submitted filename: Response to Reviewer Comments_PPATHOGENS.docx [file ppat.1009445.s013.docx]

Response to Reviewer Comments	PPATHOGENS-D-21-00460

**Part II – Major Issues: Key Experiments Required for Acceptance**
Please use this section to detail the key new experiments or modifications of existing experiments that should be absolutely required to validate study conclusions.

Generally, there should be no more than 3 such required experiments or major modifications for a "Major Revision" recommendation. If more than 3 experiments are necessary to validate the study conclusions, then you are encouraged to recommend "Reject".

Reviewer #1: (No Response)

Reviewer #2: None

Reviewer #3: The authors state in the discussion that “The key finding of this paper is that naturally acquired immunity protects from Cryptosporidium diarrhea but does not provide sterilizing immunity”, but the results do not show that increased immunity leads to less diarrhea-all that is shown is that increased immunity leads to less growth faltering. Can the relationship between IgA levels and repeat infections be analyzed and described?

Response:

The text has been modified to include the lines below information (line 294)

In children with high fecal anti-Cp23 IgA there was an increase in the number of days until a child was reinfected and a subsequent decrease in the proportion of children in this group reinfected during years 2-3 ( in children with high fecal anti-Cp23 IgA 77.8% were reinfected versus 92.2%)

In the analysis of the samples, parasite burden has been quantified by qPCR, but only the Cq of the first positive sample is used in the analysis. Is there any way to use the qPCR results to estimate total parasite burden over the 3 years?

Response:

Given the information we currently have on hand we do not feel confident that we are able to accurately estimate the total parasite burden in each child. The goal of the current study was to capture at least one positive sample per infection not to repeatedly sample any given infection. From preliminary results we were able to see that after children were infected asymptomatic cartridge could be quite variable [7-65 days]. During this time the parasite burden did not remain constant. We feel that additional studies with a greater sampling frequency are needed to adequately assess this parameter. We have added Fig S4G to provide more detail on the possible variation in parasite burden over the course of an infection.

It is not stated in the methods the timing of sample collection during diarrheal episodes-were diarrheal samples collected and tested daily? Is it possible to quantify parasite burden of a diarrheal episode by area under the curve analysis?

Response:

Diarrhea was defined as ≥3 loose stools within a 24-hour period as reported by the child’s caregiver with episodes separated by a gap of at least 3 days. The duration of diarrhea was not significantly different in the higher anti-Cp23 IgA during years 2-3 (5.35 ± 4.3 days versus 4.85 ± 3.45) . We have modified the text to add this information

**Part III – Minor Issues: Editorial and Data Presentation Modifications**
Please use this section for editorial suggestions as well as relatively minor modifications of existing data that would enhance clarity.

Reviewer #1: Minor comments and corrects:
The introduction is rather brief.
The authors might briefly specify the definition of a repeat infection in the results section (it is well described in the M&M) as this is an important feature of the experimental set up.

Response:

Our definition of a repeat infection has been added into the results section (line 78)

The investigators use value of the first detectable sample as the ‘amplitude’ of burden. Why did they do that and not something like area under the curve?

Response:

As discussed above repeated sampling during a single infection was not routinely done during this study. Frequently only one sample was captured.

I feel it may help the reader to understand this infection and the study design and results better, to see several ‘representative’ children over the entire time with all samples, essentially cv burden curves over the three years highlighting examples of the ‘epidemiological phenotypes’ they discuss later. Either in fig. 1 or as a supplementary figure.

Response:

A Supplementary figure 4 has now been included in the manuscript which includes representative infants from the high anti-Cp23 IgA group and ones which had low antibody levels. To illustrate both the variation in prasite burden and the gradual decline in parasite burden over the course of an infection a panel showing the Cq values obtained from later time points is included in this figure.

L58 cut ‘were’

Response:

Done

Reviewer #2: Table 2. This table is not very clear and overall distracts from the points. I think the manuscript would be stronger with it deleted.

Response:

We respectfully disagree with the reviewer on this point and feel that this table is a valuable addition to the manuscript. The text has been modified to make its significance clear.

Fig 3 and Fig S4. Some of the. points noted have a cQ of 40, which barely meets the case definition. Please check that these were in fact positive.

Response:

These samples are < 40 and meet the case definition

Fig 5. This figure duplicates data in the top and bottom half of the figure. Both are not needed.

Response:

The figure has been modified

Fig 6 would be clearer if labeled IgA high and low rather than group 1 and 2.

Response:

The figure has been modified

Reviewer #3: Fig 1: please clarify “within 65 days of previous”

Response:

The figure has been altered to " DS&MS samples collected within a 65 day window were grouped as a single infection"

Table 2 does not show Cq values, but the way the text is worded in lines 93-94 makes it sound like the table does have Cq values. Please re-word to make the text clearer.

Response:

The text has been modified to state

"To investigate if the reduced severity of clinical disease in recurrent infections could be correlated with a reduction in parasite burden, the amount of sub-clinical and diarrheal disease were measuredin recurrent infections were compared (Table 2)."

In many figures the mean and error bars cannot be seen (ie, Fig 2B, Fig S6 and Fig 6). Please use a different color for the bars.

Response:

Done

Fig 5A. Is the bar to the right of the table supposed to be color coded?

Response:

Panel 5A is now deleted as suggested above

Figure S5 A-D need to be enlarged.

Response:

The figure (now S6) is now altered

Line 162-Should the referral to Table 5 be instead a referral to Figure S5?

Response:

As both table 5 and the now Fig S6 illustrate this the text has been amended to refer to both

In lines 186-187 the text states the Pearson coefficient and p value for the Cryptosporidium infections: HAZ at year 3 comparison as: coef = -0.18, p=0.024. But in lines 193 to 195 the text states: ”However, while Cryptosporidium infections (diarrheal and sub-clinical) were significantly associated with child HAZ at year 3 (Pearson’s correlation p=0.0004). These statements seem to report different p values for the same comparison. Please clarify.

Response:

The former refers to the p values refer to the initial correlation between *Cryptosporidium* and HAZ (done by use of simple linear regression). In the latter case Pearson's correlation was used. The text has been amended to make this clear.

Line 196: Should say Table S2.

Response:

Thank you this has been corrected

Lines 211-214 and Figure S9A. Can you please clarify the reason why Group 2 at year 1 is split out into uninfected (2a) and pcr+ (2b) children? What does “diagnostic qPCR positive only” mean? It is unclear what children are included in group 2b. Was infection diagnosed by other methods?

Response:

Children with high levels of fecal anti-Cp23 IgA were diagnosed as being previously exposed to the Cryptosporidium parasite even if the parasite had not been identified during the PCR assay. Antibodies against Cp23 were however not always developed in response to a *Cryptosporidium* infection. In addition to the analysis shown in Fig 6 in Fig S10 the data was examined to determine if the Children who were exposed to the parasite (as determined by the detection of *Cryptosporidium* DNA by qPCR) but who did not have an antigenic response experienced more growth faltering after a *Cryptosporidium* infection than immunologically naive children.

The text has been modified to make this distinction clear.
